# Surgery and the Doctrine of Evolution

**Published:** 1882-09

**Authors:** 


### SURGERY AND THE DOCTRINE OF EVOLUTION.

C. Pitfield Mitchell, M.R.C.S., of Orange, N. J., contributes to the *New York Medical Journal and Obstectrical Review* for September, 1882, an Essay, entitled, "An Evolution Aspect of the Healing of Wounds, with Deductions as to Treatment." As the author tells us in a prefatory statement, this is an applieation of a Spencerian doctrine of evolution to some of the phenomena of reparative action. The essay sets out with a classification of methods of repair from the standpoint adopted. Next, the grounds for this classification are given, and incidentally we are introduced to an important conception—arguing that, since whatever is profitable to an organism, in the way of structural or functional variation, will be taken advantage of by heredity and natural selection, the functional changes naturally involved in recovery from disease will come within the sphere of their operations. With the zymotic diseases, for instance, natural selection may segregate, and heredity may fix both the physiological peculiarity which insures immunity, and the physiological activities which establish the *status quo* when the disease has been contracted. Entering upon the immediate topic of the paper, the phenomena elicited by an incised wound, as the occlusion of arteries, the organization of plastic lymph, the development of granulations, and the physiological adjustment of the tissues to new external conditions, are viewed as non-specific functions of the tissues injured superadded to their specific functions. Deducing the evolution of these phenomena from the known action of physical forces, the shares taken by natural selection and sexual selection as factors are then dwelt upon. Special attention is directed to the protective value of the plastic lymph forming on the surfaces of wounds, and the evolutional steps are described by which this function is acquired. Passing from the structural, the evolution of certain more strictly functional adaptations is next considered. Knowing, in general terms, the atmospheric and other forces to which wounded tissues in the past must have been exposed, the corresponding accommodations of function are inferred. Thus, the general conclusion is drawn that "the molecular constitution of wounded tissues should fit them, on the average, for contact with a mean atmosphere, and certain moderate deviations from this mean." It is pointed out that, although traumatic injuries are not necessary accompaniments of life, they are of such frequency among the lower animals and man as to give validity to this conclusion. An absence of organized adjustments of function to the remaining forces commonly incident upon wounds is inferred from the inconstancy, diversity and nature of these forces. Such deductions are shown to harmonize with experience, and certain principles of treatment for healthy wounds are presented as corollaries. The gist of them is, that so far as the exigencies of practice will permit, wounds should be shielded from the incidence of any force to which we may know *a priori* there cannot exist an organized adaptation. A normal atmosphere should be maintained, and cleanliness should be absolute at every step. Believing that the plasma exuding from the severed tissues is, by "its chemical and mechanicai properties, and contact with e vironing forces during evolutionary time, specially fitted to protect the less stable cells which lie underneath," much importance is attached to the preservation of its integrity. "Wounds should remain open until the surfaces have become glazed, and all interfering applications should be scrupulously withheld." Finally, a verification of these inferences is found in the facts disclosed by Dr. McVail, in his paper in the *British Medical Journal*, for July 23, 1882, on the results of "Ten Years' Surgery in Kilmarnock Infirmary." The method of dressing employed (dry dressing) essentially fulfilled the above-mentioned theoretical requirements, and gave, on comparative analysis, the "best general results covering a lengthened period of time that have ever been recorded in the history of British hospital surgery;" and the last group of cases reported—numbering 421, including 90 operations, 23 major amputations, 45 injuries, 52 abcesses and 7 compound fractures—showed not a single fatality from any cause.

